# The nearly complete mitochondrial genome of *Colochela zhongi* Wei, 2016 (Hymenoptera: Tenthredinidae) and phylogenetic analysis

**DOI:** 10.1080/23802359.2020.1820393

**Published:** 2020-09-16

**Authors:** Duo Wu, Hannan Wang, Meicai Wei, Gengyun Niu

**Affiliations:** a College of Life Sciences, Jiangxi Normal University, Nanchang, P. R. China; b Key Laboratory of Cultivation and Protection for Non-Wood Forest Trees, Central South University of Forestry and Technology, Ministry of Education, Changsha, P. R. China

**Keywords:** Mitogenome, next-generation sequencing, phylogeny, *Tenthredinidae*, Colochela

## Abstract

We assembled the near-complete mitochondrial genome of *Colochela zhongi* Wei, 2016 with next-generation sequencing (NGS). The total genome size of *C*. *zhongi* was 15,095 bp with 80.6% A + T content, containing 13 protein-coding genes (PCGs), 22 *tRNA* genes, and two *rRNA* genes. Three *tRNA* genes rearrangements were found compared to the ancestral organization. Phylogenetic analysis results based on heterogeneity models of 46 Symphytan and two Apocritan strongly supported that *C*. *zhongi* was closely related to *Siobla xizangensis*.

*Colochela* Malaise, 1937 is a small genus of Tenthredinidae with three species published. Wei and Nie (1998) did not mention the genus within their new classification system of *Tenthredinoidea* s. str. Taeger et al. (2010) listed *Colochela* as a junior synonym of *Neocolochelyna* Malaise, 1937. Niu and Wei (2016) reestablished *Colochela* and discussed the morphological differences in detail between *Colochela* and *Neocolochelyna*. In this study, we sequenced a mitochondrial genome of *C. zhongi* (Niu and Wei 2016) and reconstructed a phylogenetic tree with other mitochondrial sequences of Symphytan species to clarify the phylogenetic position of *Colochela* within Tenthredinidae.

Specimens (CSCS-Hym-MC0061) were collected in Kaitianguan, Taibai Mountain, Shaanxi (34.00°N 107.86°E) in Jun 2017, and are obtained at the Asia Sawfly Museum, Nanchang (ASMN) repository. Genomic DNA was sequenced by the high-throughput Illumina Hiseq 4000 platform, a total of 89,245,894 raw reads (SRR12064870) were yielded. The Bioproject number is PRJNA589586. DNA sequences were assembled using two different approaches to ensure the accuracy. In the results generated by MitoZ (Meng et al. 2019), there were assembly errors in both sequence of *nad3* and *nad5*, because a stop codon appears in each of the sequence. Then we did a parallel assembly using *Tenthredo tienmushana* (KR703581), *Siobla sturmii* (unpublish), as references in Geneious Prime 2019.2.1 (https://www.geneious.com). The mean depth of coverage across the sequences was 34,686 and 4,490, respectively. The Control region failed to be assembled in the above two methods and in NOVOPlasty (Dierckxsens et al. 2017), which is a common phenomenon in hymenoptera, possibly due to very high AT content. This result of *nad5* corrected the previous error. But the *nad3* did not get the right result. After comparing the untranslatable sequence to the relative, we manually added one conserved site to fix the error.

Annotations were first generated in MITOS web server (Bernt et al. 2013), then corrected in Geneious if necessary. The annotation sequence of the mitochondrial genome of *C. zhongi* with a length of 15,095 bp (MT702984). Compared with the putative ancestral gene arrangement of insects, there have been two rearrangement events in the mitochondrial genome of *C. zhongi*, corresponding to the rearrangement of A + T rich region-*trnI*(+)-*trnQ*(−)-*trnM*(+) clusters to *trnM*(+)-*trnQ*(−)-A + T rich region-*trnI*(+).

The A + T content of the whole mt genome was 80.6% (42.2% A, 11.3% C, 8% G, and 38.4% T), indicating significant A + T bias. All PCGs used ATN as initiation codon, and used the most common termination codon, TAA, except for *cox3*, which stopped with TAG, and for *nad1*, which stopped with T. There were 154 intergenic nucleotides between 13 gene pairs, with the longest (66 bp) identified between the *cox1* and *trnY*. Sixteen overlapped nucleotides were also found between three gene pairs, with the longest overlaps (8 bp) identified between the *trnW* and *trnC*.

The phylogenetic tree was constructed by Bayesian analysis with PhyloBayes (Lartillot et al. 2009) based on ten unsaturated amino acids data sets (*atp8*, *nad3*, and *nad4L* were excluded) of 46 Symphytan species and two Apocritan species under MtArt + CAT models conducted on the CIPRES webserver (Miller et al. 2010) (Figure 1). *Colochela zhongi* was identified as the sister group of *Siobla xizangensis*. All related files have been uploaded to figshare (https://figshare.com/projects/Colochela_zhongi/86705).

**Figure 1.**
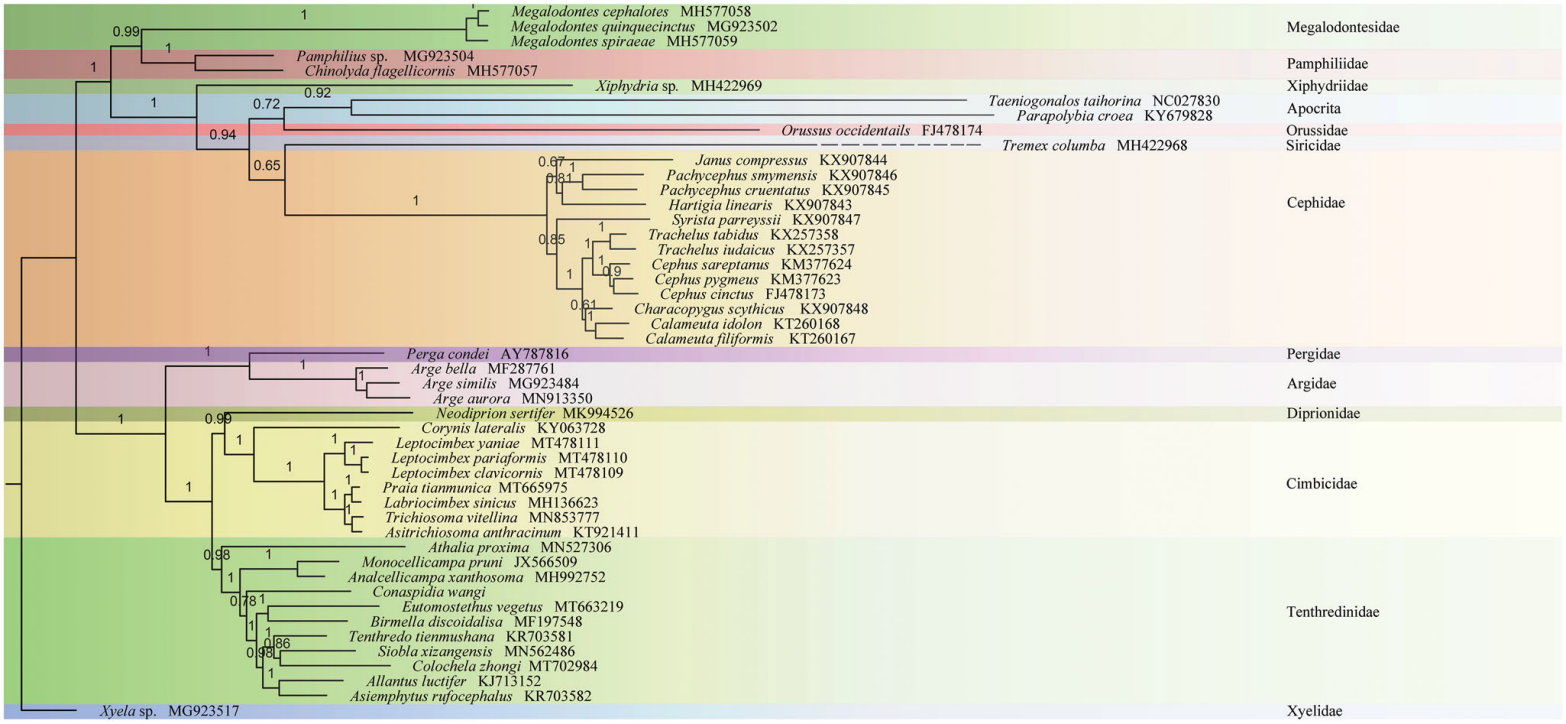
Phylobayes tree based on the combined data of 10 unsaturated amino acids. The numbers above each node are posterior probabilities. The accession number for each species is indicated after the Latin name.

## Data Availability

The data that support the findings of this study are openly available in the Figshare repository at https://figshare.com/projects/Colochela_zhongi/86705.
